# Autism and Classical Eyeblink Conditioning: Performance Changes of the Conditioned Response Related to Autism Spectrum Disorder Diagnosis

**DOI:** 10.3389/fpsyt.2016.00137

**Published:** 2016-08-11

**Authors:** John P. Welsh, Jeffrey T. Oristaglio

**Affiliations:** ^1^Center for Integrative Brain Research, Seattle Children’s Research Institute, Seattle, WA, USA; ^2^Department of Pediatrics, University of Washington Autism Center, University of Washington, Seattle, WA, USA; ^3^Department of Pharmacology and Physiology, Drexel University College of Medicine, Philadelphia, PA, USA

**Keywords:** autism, eyeblink conditioning, timing, cerebellum, diagnostic specificity

## Abstract

Changes in the timing performance of conditioned responses (CRs) acquired during trace and delay eyeblink conditioning (EBC) are presented for diagnostic subgroups of children having autism spectrum disorder (ASD) aged 6–15 years. Children diagnosed with autistic disorder (AD) were analyzed separately from children diagnosed with either Asperger’s syndrome or Pervasive developmental disorder (Asp/PDD) not otherwise specified and compared to an age- and IQ-matched group of children who were typically developing (TD). Within-subject and between-groups contrasts in CR performance on sequential exposure to trace and delay EBC were analyzed to determine whether any differences would expose underlying functional heterogeneities of the cerebral and cerebellar systems, in ASD subgroups. The EBC parameters measured were percentage CRs, CR onset latency, and CR peak latency. Neither AD nor Asp/PDD groups were impaired in CR acquisition during trace or delay EBC. Both AD and Asp/PDD altered CR timing, but not always in the same way. Although the AD group showed normal CR timing during trace EBC, the Asp/PDD group showed a significant 27 and 28 ms increase in CR onset and peak latency, respectively, during trace EBC. In contrast, the direction of the timing change was opposite during delay EBC, during which the Asp/PDD group showed a significant 29 ms decrease in CR onset latency and the AD group showed a larger 77 ms decrease in CR onset latency. Only the AD group showed a decrease in CR peak latency during delay EBC, demonstrating another difference between AD and Asp/PDD. The difference in CR onset latency during delay EBC for both AD and Asp/PDD was due to an abnormal prevalence of early onset CRs that were intermixed with CRs having normal timing, as observed both in CR onset histograms and mean CR waveforms. In conclusion, significant heterogeneity in EBC performance was apparent between diagnostic groups, and this may indicate that EBC performance can report the heterogeneity in the neurobiological predispositions for ASD. The findings will inform further explorations with larger cohorts, different sensory modalities, and different EBC paradigms and provide a reference set for future EBC studies of children having ASD and non-human models.

## Introduction

The purpose of this paper is to describe the changes in conditioned response (CR) performance during trace and delay eyeblink conditioning (EBC) in a cohort of high-functioning children having autism spectrum disorder (ASD). The *de novo* acquisition and mean parameters of CR timing of this cohort of 14 children with ASD and 16 typically developing (TD) children were reported by Oristaglio et al. (1). Here, we provide more detailed information regarding the distribution of CR performance changes in the cohort described by Oristaglio et al. (1), re-grouped by ASD-spectrum diagnosis in order to determine whether heterogeneity in EBC performance may relate to diagnostic category within a high-functioning group of children. Our experimental design sequentially employed trace and delay EBC paradigms in every subject to provide within-subject and between-group contrasts of EBC performance on two paradigms that differ in their degree of cerebral involvement (2).

Two previous studies (1, 3) of subjects having idiopathic ASD demonstrated changes in CR timing during delay EBC without a reduction in the rate of CR acquisition, agreeing that CR timing is shifted earlier with ASD. In a complementary pair of studies (4, 5), delay EBC in children and adults with Fragile X, a severe form of intellectual disability in which some individuals have comorbid symptoms of ASD, showed reduced CR acquisition and earlier CR peak latencies with greater impairments in adults as compared to children. Thus, four studies have demonstrated heterogeneity in the changes in EBC performance among individuals with varying ASD symptomatology. Although not explicitly emphasized, this heterogeneity is mirrored in mouse models of idiopathic and syndromic ASD in which there are differential changes in the rate of CR acquisition and the directionality of CR timing that depend on the specific gene mutation induced (4, 6, 7).

For our study, the rationale for using a sequence of trace and delay EBC was to provide the first examination of trace EBC in children with ASD, followed by an opportunity to determine whether we could replicate the previous finding of mistimed CRs during delay EBC. Although the division of the original cohort in Oristaglio et al. (1) into subgroups necessarily reduces group size and statistical power, our goal is to provide these data so that they may serve as a reference for future studies of EBC using larger cohorts of children having ASD with varying degrees of functional impairment and for those that use classical conditioning of the eyeblink or other responses in non-human animal models of ASD.

## Materials and Methods

### Subjects

The subjects were 30 children (age 6–15 years) recruited at the Drexel Autism Center at Friends Hospital in Philadelphia. The study was approved by the IRB of the Drexel University College of Medicine and a legal custodian of the subjects signed a consent form prior to participation. Fourteen subjects were diagnosed with ASD (13 males, 1 female), and 16 were typically developing (TD; 7 males, 9 females). ASD subjects included those diagnosed with autistic disorder (AD; *n* = 7), Asperger’s disorder (Asp, *n* = 2), and pervasive developmental disorder-not otherwise specified (PDD-NOS, *n* = 5) based on the content-area scores on the revised Autism Diagnostic Interview [ADI-R; Ref. (8)] and the Childhood Autism Rating Scale (9). By convention, children with Asp or PDD-NOS met criteria on two of the three domains assessed on the ADI-R and showed developmental delays prior to 3 years of age based on retrospective report. Exclusion factors for the ASD group were the presence of psychiatric diagnosis including Rett’s disorder or childhood disintegrative disorder. TD subjects had no psychiatric diagnoses other than one diagnosed with oppositional defiant disorder and obsessive-compulsive disorder. The ASD and TD subjects were approximately matched for age [mean ± 1 SEM: TD, 9.6 ± 2.5; AD, 7.7 ± 1.3; Asp/PDD, 9.4 ± 0.6 years; *F*(2,27) = 1.4, *p* = 0.3] and IQ [TD, 111 ± 3; AD, 107 ± 3; Asp/PDD, 104 ± 5 WASI score; *F*(2,27) = 0.8, *p* = 0.5].

### Eyeblink Conditioning

Eyeblink conditioning was carried out as specified in Oristaglio et al. (1). Briefly, the subjects watched a silent movie while they wore headphones that delivered tones binaurally. Eye blinks were detected by an infrared emitter-sensor approximately 1 cm from the right eye. Eye blinks were defined as a change in sensor output greater than 15 SD above the mean baseline. The conditioned stimulus (CS) was a 1-kHz, 61-dB tone. The unconditioned stimulus (US) was a 100 ms puff of air (5 psi source) delivered to the eye through a tube (1 mm i.d.) attached to the sensor. EBC sessions contained 90 trials divided into nine blocks of 10 trials. The first nine trials in each block consisted of paired CS–US trials, and the 10th was a CS-alone trial. The intertrial interval was 20 s (range 15–25 s). Three EBC sessions occurred on separate visits. The first two sessions consisted of trace EBC, which was performed using a 200-ms CS, a 500-ms trace interval, and then the US (700 ms CS–US interval). The third session consisted of delay EBC also at the 700 ms CS–US interval and was performed by extending the CS duration so that it coterminated with the US. CRs were defined as eye blinks that occurred at least 80 ms after CS onset and prior to US onset. This experimental design used a constant CS–US interval in order to place no explicit motor demand on CR timing while changing only the EBC paradigm from trace to delay on session 3. Although the two paradigms would necessarily interact, there is precedent for using sequences of delay and trace EBC within the same human subjects in clinical studies (10, 11). Moreover, functional brain imaging has demonstrated differential brain activation in humans experiencing both paradigms concurrently (2).

The mean time between the two trace EBC sessions was 13 ± 2 days, and the mean time between the second trace EBC session and the delay EBC session was 27 ± 9 days. There was not a difference between the groups in the number of days between sessions for between the trace sessions [*F*(2,27) = 1.7, *p* = 0.2] or between the trace and delay sessions [*F*(2,27) = 1.0, *p* = 0.4].

### Statistical Analysis

Conditioned response acquisition was examined using mixed effects analysis of variance. Differences in CR performance were examined with three methods using the post-CS response distributions as primary data. First, differences in the means of the individual responses were evaluated by paired *t*-test. Second, differences in CR distributions were evaluated using the non-parametric, two-sample Kolmogorov–Smirnov (K–S) test, a highly liberal test that evaluates whether there is a difference in the shape of the cumulative probability function at any unspecified location in the CS–US interval. Third, differences between medians were evaluated using the non-parametric Mood’s median test. Mood’s median test evaluated whether there is a difference in the ratio of responses below and above an aggregate median, which is tested using a 2 × 2 contingency table. Mood’s median test was chosen due to its robustness against differences in the shapes of the latency distributions between groups. The three tests were employed under the rationale that the most significant effects of ASD diagnosis would produce a significant change not only in the cumulative probability function but also in the more conservative mean and median tests that inform about the directionality of a change in central tendency. We labeled CR performance changes as strong or moderate depending on the number of statistical tests that detected a difference between the distributions. CR waveform analysis was carried out by averaging CRs triggered on CS onset. Last, mean CR onset and peak latency for each individual subject was *z*-transformed using the TD mean and SD and presented for each EBC session. The threshold used for statistical significance was 5%. Data are presented as the mean ± 1 SEM.

## Results

**Figure 1:**
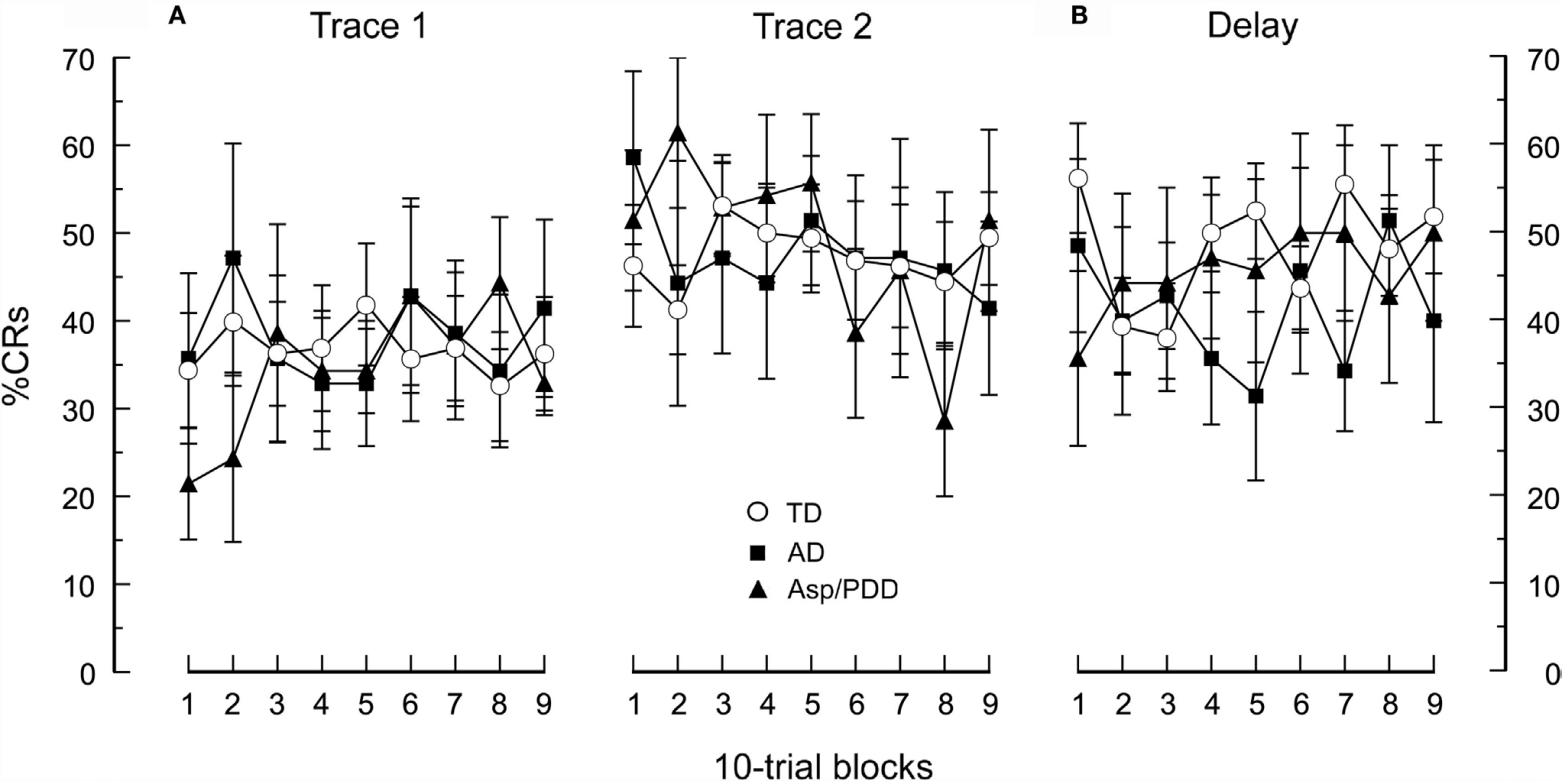
**CR acquisition for the AD, Asp/PDD, and TD groups**. **(A)** During two sessions of trace EBC. **(B)** During a subsequent session of delay EBC. Mean data are presented in 10-trial blocks. Error bars show ± 1 SEM. Curves showing TD are taken from Oristaglio et al. (1).

As reported for this cohort (1), the rate of CR acquisition and asymptotic percentage CRs did not differ between the 14 ASD and 16 TD subjects during any of the three EBC sessions. Figure 1 shows the percentage CRs over sessions in which the ASD cohort was divided into diagnostic subgroups. All groups showed associative learning by displaying a significant increase in percentage CRs from the first to the second session [*F*(1,17) = 3.1, *p* < 0.005]. There was not a significant difference in learning rate between the groups [*F*(1,27) = 0.01, *p* = 0.99] nor a significant difference in the shape of the learning curves across the trace EBC sessions [*F*(1,17) = 0.8, *p* = 0.67; Figure 1A]. Switching to delay EBC did not significantly change the percentage CRs from the previous session (Figure 1B), and there was no significant difference between groups as the overall mean CR frequency during delay EBC was 48 ± 4, 41 ± 7, and 46 ± 8% for TD, AD, and Asp/PDD, respectively [*F*(2,27) = 0.1, *p* = 0.93 for the first three blocks; *F*(8,216) = 0.5, *p* = 0.63 for all nine blocks]. Thus, there was no indication that AD or Asp/PDD impaired the ability to acquire CRs during trace or delay EBC or show asymptotic percentage CRs characteristic of the TD group under these EBC parameters.

**Figure 2:**
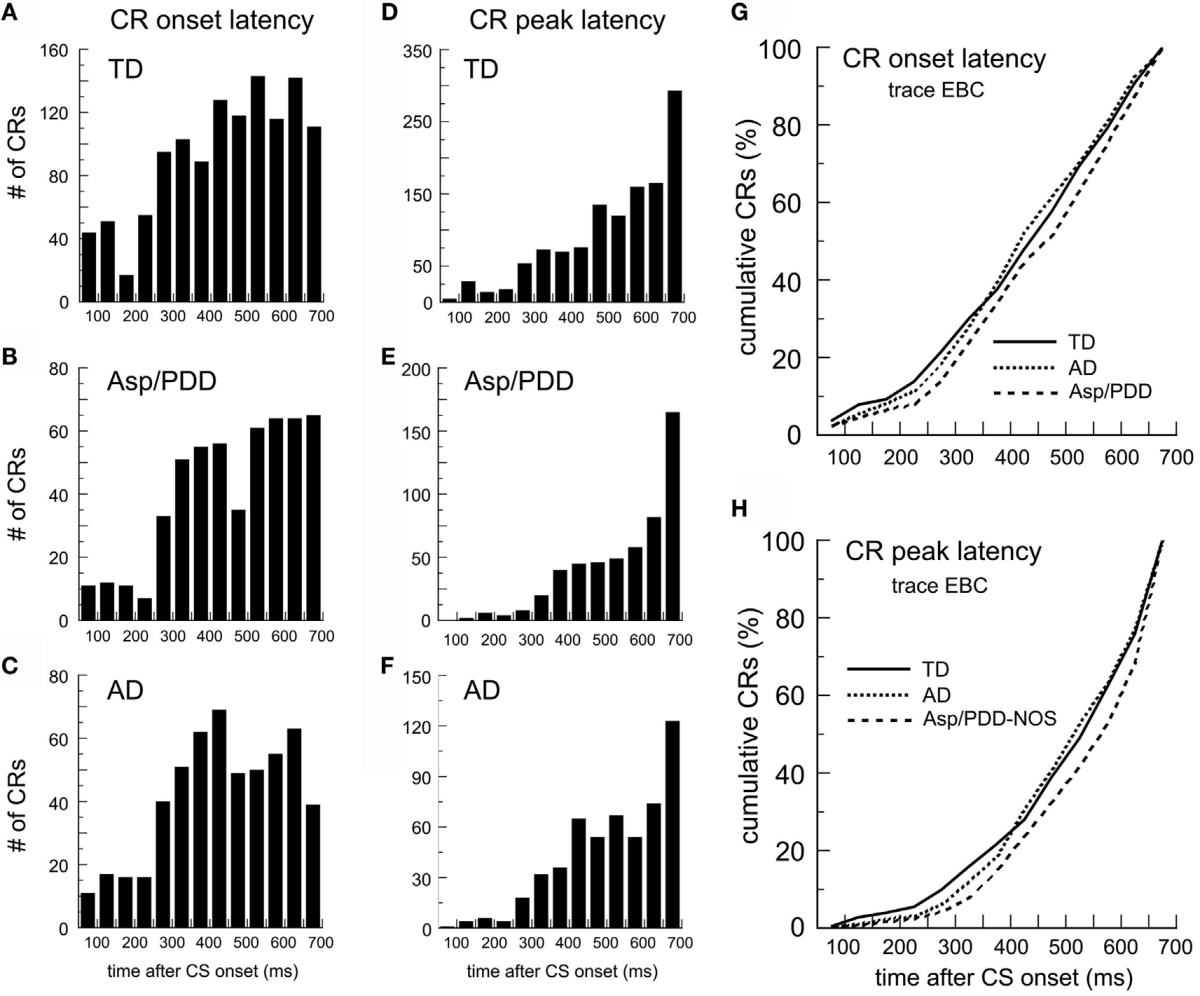
**CR timing during trace EBC**. **(A–C)** Post-CS time histograms of CR onset for the TD **(A)**, Asp/PDD **(B)**, and AD **(C)**. **(D–F)** CR peak latency histograms from the same groups. **(G,H)** Cumulative probability plots of CR onset and CR peak. Bin width = 50 ms. Time 0 = CS onset.

Figure 2 shows post-CS time histograms and cumulative probability plots of CR onset and peak latency over two sessions of trace EBC. The mean CR onset latencies were: TD, 441 ± 5; AD, 441 ± 7; Asp/PDD, 468 ± 7 ms. *T*-tests did not indicate a difference between TD and AD [*t*(1,748) = 0.09, n.s.] but did indicate a significant difference between TD and Asp/PDD [*t*(1,735) = 3.1, *p* < 0.01]. K–S tests indicated that the shape of both the AD (*D* = 0.04, *p* < 0.01) and Asp/PDD (*D* = 0.08, *p* < 0.001) distributions differed from TD. However, Mood’s median test did not detect a significant difference of the median CR onset latency of either diagnostic group when compared to TD (both *p* ≥ 0.1). Thus, there was a moderate indication of a small delay in CR onset latency during trace EBC for Asp/PDD (27 ms), but not AD.

The mean CR peak latencies during trace EBC were TD, 508 ± 4; AD, 518 ± 6; and Asp/PDD, 536 ± 6 ms. Again, *t*-tests did not indicate a difference between TD and AD [*t*(1,643) = 1.31, n.s.], but did indicate a significant difference between TD and Asp/PDD [*t*(1,592) = 3.6, *p* < 0.01]. K–S tests indicated that the shape of both the AD (*D* = 0.04, *p* < 0.05) and Asp/PDD (*D* = 0.09, *p* < 0.01) distributions differed from TD. However, Mood’s median test did not detect a significant difference between either diagnostic group and TD, although Mood’s test between TD and Asp/PDD was nearly significant (X^2^ = 3.79, *p* = 0.052). Thus, as for CR onset latency, there was a moderate indication of a small increase in CR onset latency during trace EBC for Asp/PDD (28 ms), but not for AD.

**Figure 3:**
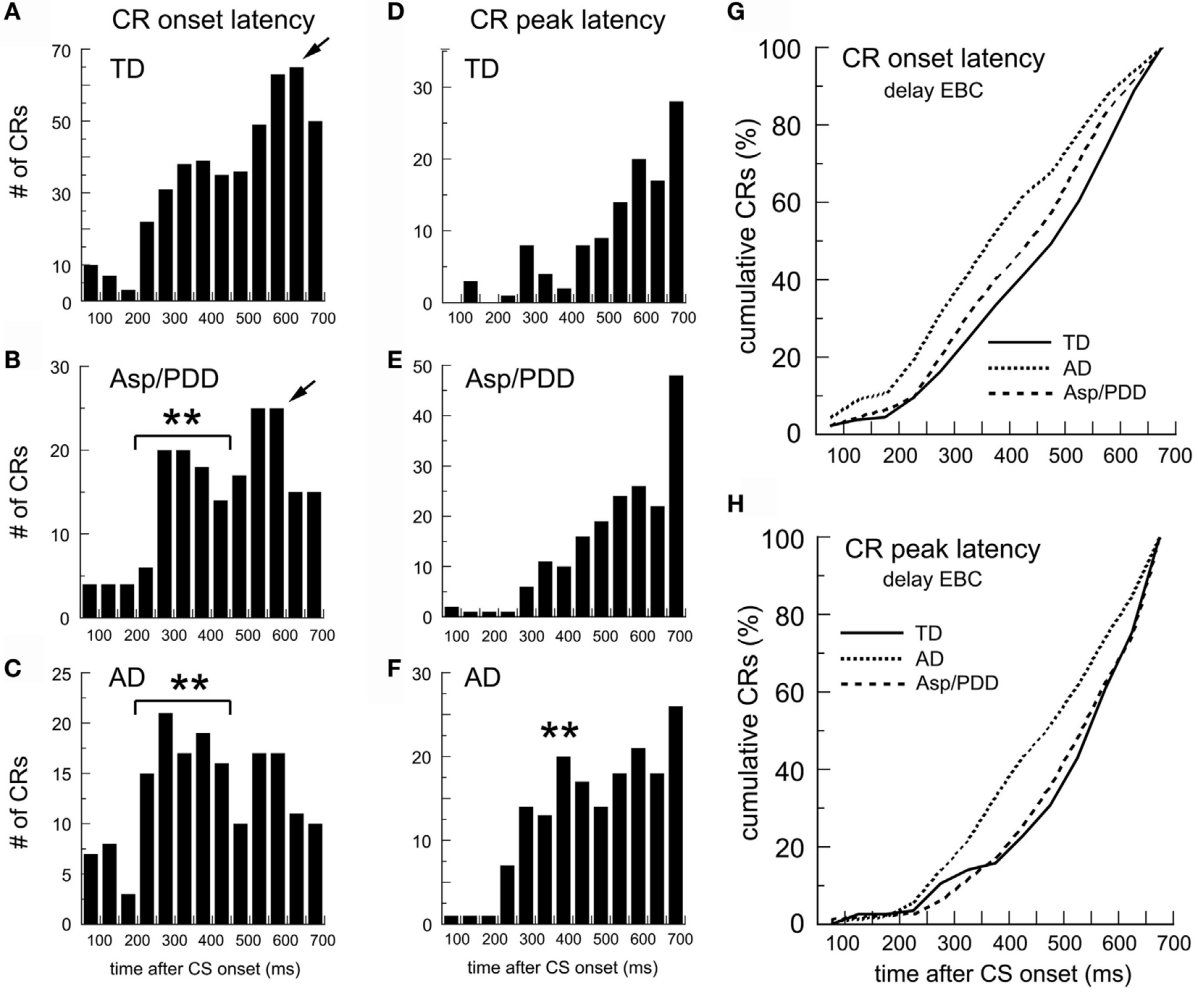
**CR timing during delay EBC**. **(A–C)** Post-CS time histograms of CR onset. **(D–F)** CR peak latency histograms. **(G,H)** Cumulative probability plots of CR onset and CR peak. Bin width = 50 ms. Time 0 = CS onset. Arrows show mode onsets. Asterisks indicate early CRs.

Figure 3 shows the identical analysis for delay EBC. The mean CR onset latencies were TD, 472 ± 7; AD, 395 ± 13; and Asp/PDD, 443 ± 11 ms. *T*-tests indicated a highly significant difference between TD and AD [*t*(617) = 5.4, *p* < 0.001] and a significant difference between TD and Asp/PDD [*t*(633) = 2.2, *p* < 0.05]. The CR onset latency histogram of the TD group was shaped such that the prevalence of CR onsets increased as the CS–US interval progressed, reaching maximum 650 ms after CS onset (Figure 3A, arrow). In contrast, AD subjects showed a mode CR onset at 300 ms, and the majority of their CRs were initiated between 200 and 400 ms after CS onset (Figure 3C, asterisks). The distribution of the Asp/PDD group had two peaks, the first identical to AD between 200 and 400 ms (Figure 3B, asterisks) and the second close to the TD peak at 600 ms (Figure 3B, arrow). K–S analyses indicated that the changes in distribution shapes were highly significant (AD vs. TD: *D* = 0.21, *p* < 0.001; Asp/PDD vs. TD, *D* = 0.11, *p* < 0.001). Mood’s median test detected a highly significant difference between the CR onsets of TD and AD (X^2^ = 20.8, *p* < 0.001) but not between TD and Asp/PDD (X^2^ = 3.3, n.s.). Thus, there was a strong indication of a large decrease in CR onset latency for AD (77 ms) and a moderate indication for a smaller decrease for Asp/PDD (29 ms).

The mean CR peak latencies during delay EBC were: TD, 544 ± 13; AD, 482 ± 11; Asp/PDD, 536 ± 10 ms. *T*-tests indicated a highly significant difference between TD and AD [*t*(282) = 3.6, *p* < 0.001], but not between TD and Asp/PDD [*t*(298) = 0.4, n.s.]. K–S analysis indicated a highly significant difference between TD and AD (*D* = 0.21, *p* < 0.001), but not between TD and Asp/PDD (*D* = 0.06, n.s.). Mood’s median test also detected a highly significant difference between the CR peak latencies of TD and AD (X^2^ = 10.2, *p* < 0.01), but not between TD and Asp/PDD (X^2^ = 1.4, n.s.). Thus, there was a strong indication of a decrease in CR peak latency for AD (62 ms), but no indication of a change for Asp/PDD.

Table 1 presents the outcomes of the above comparisons. In sum, there was moderate indication that Asp/PDD, but not AD, was associated with a small increase in CR onset and peak latency during trace EBC. There was a strong indication that subjects with AD had significantly reduced CR onset and peak latencies during delay EBC. Reduced CR onset latencies during delay EBC were also observed in the Asp/PDD group, but to a smaller degree, and, unlike the AD group, the CR peak latency was not significantly different for Asp/PDD during delay EBC.

**Table 1:** **Magnitude and direction of CR performance changes for the AD and Asp/PDD groups**.

	CR onset latency			CR peak latency		
	K–S	*t*-test	Median	K–S	*t*-test	Median
Trace						
AD	**Yes	No	No	*Yes	No	No
Asp/PDD	***Yes	**Yes ↑	No	**Yes	**Yes ↑	No
Delay						
AD	***Yes	***Yes ↓↓	***Yes ↓↓	***Yes	***Yes ↓↓	**Yes ↓↓
Asp/PDD	***Yes	*Yes ↓	No	No	No	No

*Arrows indicate directionality of change (up arrow = increased latency; down arrow = decreased latency), relative to the TD group*.

*Color indicates the statistical strength of performance change (green = consistently positive; yellow = moderate; red = consistently negative)*.

*p < 0.05, **p < 0.01, ***p < 0.001, compared to TD.

**Figure 4:**
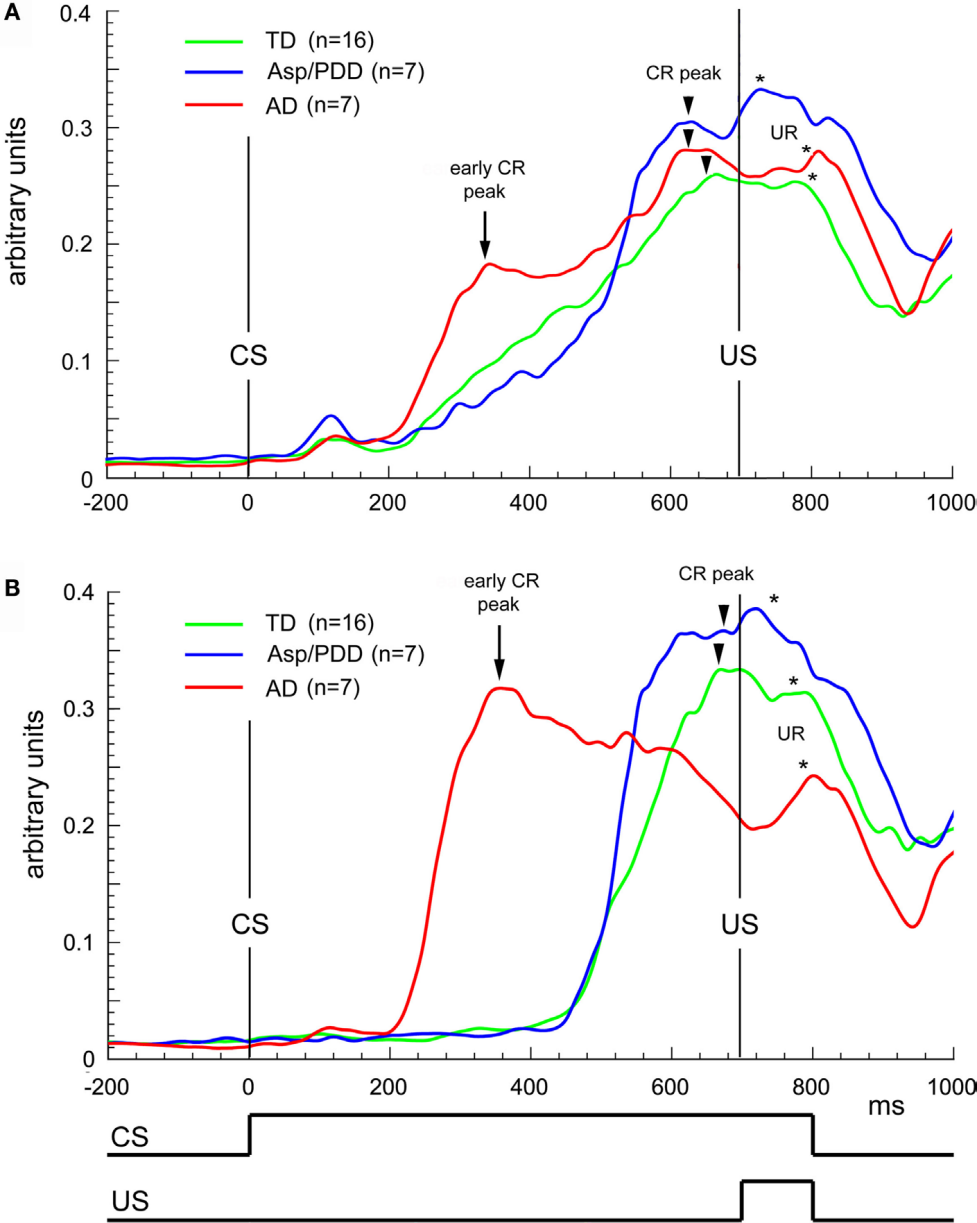
**Waveform analysis during delay EBC**. **(A)** Average CRs during the first 30 trials of delay EBC for TD (green), Asp/PDD (blue) and AD (red) groups. **(B)** Average of the most prevalent type of CR for each group. Arrows indicate abnormally early CR peaks by the AD group. Arrowheads indicate CR peak close to US onset. Asterisks indicate unconditioned response peaks to the airpuff US. Curves show the mean of all subjects in each group.

Figure 4A plots the average topography of the CRs on the first 30 trials of delay EBC. The most significant deviation from the monophasic waveform of the TD group (Figure 4A, green) was the presence of two peaks in the average CR of the AD group (Figure 4A, red), with the first peak at 350 ms (Figure 4A, arrow) and the second at 600 ms (Figure 4A, arrowhead on red trace). The average CR of the AD group also differed from Asp/PDD waveform, which also showed only a single peak at approximately 600 ms (Figure 4A, arrowheads). The two peaks in the average CR of the AD group was consistent with either a biphasic CR or the averaging of two types of CRs having early and late timing. The analysis was repeated by selecting the CRs having modal onsets within each of the groups. For the AD group, this corresponded to CRs with onsets between 200 and 450 ms (Figure 4B, red) detected in the onset histograms (Figure 3), while, for the other two groups, this corresponded to CRs with onsets between 500 and 650 ms. The early CRs of AD subjects showed only one, abnormally early peak that occurred at 350 ms (Figure 4B, arrow), thereby accounting for the first of the two peaks in the average CR and indicating that they were not biphasic CRs but rather monophasic CRs that were inappropriately timed. Notably, those early CRs did not maintain peak amplitude throughout the CS–US interval, unlike the average CR of TD and Asp/PDD that peaked within 50 ms of the US (Figures 4A,B, arrowheads).

**Figure 5:**
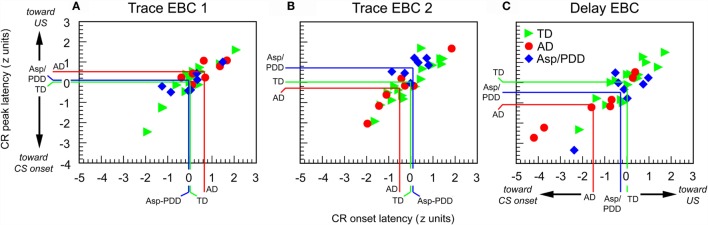
**Individual CR timing data during the first 30 trials of each EBC session**. Data are normalized to the TD group mean and expressed as standard scores. Colored lines indicate means of TD (green), AD (red), and Asp/PDD (blue) groups.

There was significant heterogeneity among the subjects with regard to CR performance. Figure 5 shows plots of normalized values of CR onset latency vs. peak latency relative to the TD mean for every subject. It can be seen that the three groups overlapped on session 1, which was trace EBC (Figure 5A), and largely overlapped on session 2, which was also trace EBC (Figure 5B). Of note was that three of seven AD subjects during the second trace EBC session moved into the lower half of the TD distribution, while the Asp/PDD distribution did not shift. During session 3, which was delay EBC (Figures 5C), five of seven AD subjects separated further and fell below both the TD (Figure 5C, green lines) and Asp/PDD (Figure 5C, blue lines) means for both CR onset and peak latency, with two AD subjects far outside the TD range. During delay EBC, the mean deviations of CR onset and peak latency were 1.5 and 1.1 SDs from the TD mean, respectively (Figure 5C, red lines).

## Discussion

There are two reports in the literature that have described alterations in classical EBC in high-functioning subjects with ASD (1, 3). Both reports used a heterogeneous subject pool, either due to a wider age range than is standard in current ASD research (3) and/or due to the pooling of current diagnostic categories within the ASD spectrum. Here, we reexamined the data presented in Oristaglio et al. (1) that pooled children diagnosed with AD, Asp, and PDD-NOS into one group. By separating subjects with AD and analyzing two subgroups, this is the first description of differential effects of ASD diagnoses on CR performance in high-functioning children. A limitation of the present study is the small number of subjects within the groups. Thus, our preliminary findings warrant replication with larger populations of children along the ASD spectrum and at different ages. Advantages of EBC are the stereotypic nature of CR performance and the fact that EBC can be applied across a wide range of cognitive functioning in a standardized manner. Because EBC is a robust test of associative learning and motor timing that interrogates the functioning of the cerebral and hindbrain–cerebellar systems in trace and delay paradigms, respectively, it may have further utility for evaluating brain dysfunction in pediatric populations with ASD.

By disaggregating diagnostic groups, we determined that subjects with AD were largely responsible for the finding that ASD is related to shorter CR onset and peak latencies during delay EBC (1, 3). The sensitivity of CR performance during the early stages of delay EBC in the AD group was indicated by three statistical tests that evaluated changes in central tendency and distribution shape. The analysis provided the new finding that subjects with Asp/PDD, but not those with AD, showed a difference in motor timing during trace EBC in which both CR onset and peak latencies were delayed. The magnitude of that effect was much smaller than the reduced CR latency shown by AD subjects during delay EBC, but may have important consequences for understanding brain regions that may be differentially impacted in diagnostic subcategories of ASD. Because decreases in CR onset and peak latency during delay EBC have been associated with damage to the cerebellar cortex (12, 13), the EBC phenotype with AD is consistent with a potential cerebellar involvement in the CR performance change. Because Asp/PDD subjects showed slightly shorter CR onset latencies during delay EBC and no change in CR peak latency, this may be consistent with more subtle cerebellar involvement than in AD. Of particular relevance is the finding that there is significant heterogeneity among individuals with ASD in the loss of Purkinje cells in cerebellar cortex (14). Although the loss of Purkinje cells or other cell types in the cerebellum may not pertain to the majority of cases of ASD, changes in excitability and/or plasticity in cerebellar and pre-cerebellar neurons also may underlie CR performance changes in ASD (15), as confirmed in mouse models of tuberous sclerosis (16), Fragile X (4), and 15q11–13 duplication (6), all of which are conditions associated with ASD in humans. On the other hand, the slightly longer CR onsets that Asp/PDD subjects demonstrated during trace EBC potentially implicates an additional disruption in a telencephalic process that plays a larger role for specifying CR timing during trace EBC.

We observed that there is significant heterogeneity in CR performance within an ASD diagnostic group and that there is overlap between groups. For instance, two of seven subjects in the AD group showed normal CR timing during delay EBC, and one of seven subjects in the Asp/PDD group showed a change in CR performance as extreme as the most-affected AD subjects. The neurobiological causes of these effects remain to be elucidated, but future studies of brain morphology and neurochemistry may be helpful (17, 18).

The observation of heterogeneity in EBC performance at different points along the ASD spectrum is consistent with the contrast between high-functioning children with idiopathic ASD and individuals with Fragile X syndrome, a form of severe intellectual disability in which approximately 50% have comorbid symptoms of ASD. As two reports confirm (1, 3), high-functioning individuals with ASD are not impaired in their ability to acquire CRs, but many have CR onset and peak latencies during delay EBC that are earlier than normal. In the case of Fragile X, affected individuals similarly show earlier CR peak latencies during delay EBC, but also a prominent reduction in CR acquisition in subjects older than 45 years (4, 5). Our preliminary indication that children with Asp/PDD show an increase in CR latency during trace EBC and a small decrease in CR latency during delay EBC, while children with AD show normal CR performance during trace EBC but a large decrease in both CR onset and peak latency during delay EBC helps further indicate that there is heterogeneity in CR performance changes across the ASD spectrum.

Understanding the genetic predispositions for the magnitude and direction of EBC performance changes across the ASD spectrum will be a promising direction for future clinical studies. A recent report (7) of CR performance during delay EBC to a light CS in mouse models of idiopathic and syndromic ASD indicated that monogenetic mutations can have differential effects on CR performance. For instance, a globally expressed truncation mutation in Shank3 decreased CR peak latency by approximately 30 ms, and a truncation mutation in MeCP2 increased CR peak latency equivalently. However, global Cntnap2 knockout, 15q(11–13) duplication, and Purkinje cell-specific knockout of tuberous sclerosis protein had no effect on CR timing despite being models of human ASD. It is noteworthy that the overall magnitude of the CR performance changes in our AD group during delay EBC was much larger than observed in any mouse model produced by monogenetic deletion or mutation, which may reflect the polygenetic nature of idiopathic autism in humans that affects brain development, connectivity, and synaptic physiology to various degrees across individuals (19). Moreover, understanding the contributions that alterations in sensory processing may play in affecting EBC performance across the ASD spectrum will be important, as there is significant heterogeneity among individuals in the directionality of changes in sensitivity that can differ across sensory modalities. It could be important to determine whether the delays in tone-evoked potentials and high-frequency oscillations in the superior temporal gyrus (20) observed in children with ASD having language impairment relate to delays in CR performance during trace EBC with an auditory CS, generally believed to require greater cerebral involvement than delay EBC, as well as whether any of the effects on CR performance reported here generalize to visual or tactile CSs.

As previously discussed (1), the abnormally short-latency CRs during delay EBC in the present cohort of high-functioning children with ASD replicate the major effect reported by Sears et al. (3) in a smaller group of individuals having ASD that spanned a larger-age range. Interestingly, in both Sears et al. (3) and Oristaglio et al. (1), the changes in CR timing with idiopathic ASD were not accompanied by an impaired ability to acquire CRs. Two differences between the results of our study and Sears et al. (3) were that the latter study observed enhanced CR acquisition in subjects with ASD relative to subjects with TD and overall greater asymptotic percent CRs than in our study. Those differences may be more apparent than real, however, for six reasons. First, Sears et al. (3) employed delay EBC exclusively, while our subjects were initially trained on trace EBC, a more difficult task that typically results in more modest learning performance in both human and animal studies. Second, Sears et al. (3) employed a 350 ms CS–US interval and an intensity of a tone CS that is about double in perceived loudness, both of which are well known to increase the rate of CR acquisition and the asymptotic percent CRs as compared to the 700 ms CS–US interval and 61-dB tone employed in our study (21). We specifically chose the 700 ms CS–US interval to produce a more difficult learning paradigm that would enhance the ability to detect differences between diagnostic groups and a softer tone CS to prevent distress in the children. Third, Sears et al. (3) measured CRs with corneo-retinal potentials in contrast to our use of an infrared detector. This significant difference in method may have contributed to Sears et al.’s (3) increased detection of small amplitude CRs supported by both associative and non-associative factors. Fourth, the faster CR acquisition in Sears et al. (3) may indicate a heightened ability to process short CS–US intervals in ASD that is not apparent when a 700 ms CS–US interval is employed. That possibility is the most interesting with regard to ASD neurobiology and can be tested explicitly in the future using different CS–US intervals in a within-subject design. Fifth, we observed considerable variability in the rate of CR acquisition for children in our ASD subgroups, with some performing as well or better than TD subjects while others performing below the performance of the TD group. This may potentially be accounted for by heterogeneity in sensory processing that does not segregate according to the clinical criteria which define the diagnostic subgroups. Sixth, the significant heterogeneity in the causes and symptoms of ASD and the fact that the ASD populations of Sears et al. (3) and Oristaglio et al. (1) were ascertained by different clinicians separated by 20 years, over which time inclusion/exclusion criteria and treatment have gradually shifted, could have contributed to the differences in CR acquisition, while greatly increasing the significance of the replicated effect on CR timing. Additional clarification will require studies of much larger ASD populations than has been performed to date.

In sum, our study provides an initial examination of the utility of trace and delay EBC for distinguishing ASD subgroups and suggests differential effects and heterogeneity of CR performance between and within subgroups. This provides a reference dataset for future studies of larger populations of children and non-human animals that may examine the genetic and neurobiological bases of the directionality and magnitude of CR performance differences with ASD and whether these differences generalize to other forms of sensory-motor timing.

## Author Contributions

JW and JO analyzed the data and wrote the paper.

## Conflict of Interest Statement

The authors declare that the research was conducted in the absence of any commercial or financial relationships that could be construed as a potential conflict of interest.
